# Sulodexide Significantly Improves Endothelial Dysfunction and Alleviates Chest Pain and Palpitations in Patients With Long-COVID-19: Insights From TUN-EndCOV Study

**DOI:** 10.3389/fcvm.2022.866113

**Published:** 2022-05-12

**Authors:** Salma Charfeddine, Hassen Ibnhadjamor, Jihen Jdidi, Slim Torjmen, Salma Kraiem, Amine Bahloul, Ahmed Makni, Nesrine Kallel, Nedia Moussa, Mariem Boudaya, Imen Touil, Aiman Ghrab, Jamel Elghoul, Zeineb Meddeb, Yamina Thabet, Kais Ben Salem, Faouzi Addad, Kamel Bouslama, Sami Milouchi, Rania Hammami, Salem Abdessalem, Leila Abid

**Affiliations:** ^1^Cardiology Department, Hedi Chaker University Hospital, Sfax, Tunisia; ^2^Faculty of Medicine, University of Sfax, Sfax, Tunisia; ^3^Cardiology Department, Tahar Sfar Hospital, Mahdia, Tunisia; ^4^Preventive Medicine Department, Hedi Chaker University Hospital, Sfax, Tunisia; ^5^Pneumology Department, Hedi Chaker University Hospital, Sfax, Tunisia; ^6^Biochemistry Laboratory, Hedi Chaker University Hospital, Sfax, Tunisia; ^7^Pneumology Department, Tahar Sfar Hospital, Mahdia, Tunisia; ^8^Cardiology Department, Habib Bourguiba Hospital Medenine, Medenine, Tunisia; ^9^Pneumology Department, Habib Bourguiba Hospital Medenine, Medenine, Tunisia; ^10^Internal Medicine Department, Mongi Slim LaMarsa Hospital, Tunis, Tunisia; ^11^Private Doctor, Tunis, Tunisia; ^12^Clinic Hannibal Tunis, Tunis, Tunisia; ^13^Clinic Pasteur Tunis, Tunis, Tunisia

**Keywords:** COVID-19, sulodexide, long COVID-19 syndrome, endothelial dysfunction, microcirculation

## Abstract

**Objective:**

Non-respiratory long-coronavirus disease 2019 (COVID-19) symptoms are mainly related to a long-lasting endothelial dysfunction and microcirculation impairment. We hypothesized that Sulodexide, a purified glycosaminoglycan mixture with a beneficial endothelial effect in arterial and venous peripheral diseases, may be effective in a subset of patients with long COVID-19.

**Approach and Results:**

We conducted a multicenter prospective quasi-experimental study. A total of 290 patients from the TUN-EndCOV study with long-COVID-19 symptoms and endothelial dysfunction were included. The endothelial function was clinically assessed using a post-occlusive reactive hyperemia protocol with finger thermal monitoring device. Endothelial quality index (EQI) was assessed at inclusion and at 21 days later. The study population was assigned to a sulodexide group (144 patients) or a no-medical treatment group (146 patients). Clinical characteristics were similar at inclusion in the two groups. Fatigue, shortness of breath, and chest pain were the most common symptoms, respectively, 54.5, 53.8, and 28.3%. At 21 days, the sulodexide group improved significantly better than the no-medical treatment group in chest pain (83.7 vs. 43.6%, *p* < 10^−3^), palpitations (85.2 vs. 52.9%, *p* = 0.009), and endothelial function [median delta-EQI 0.66 (0.6) vs. 0.18 (0.3); *p* < 10^−3^]. Endothelial function improvement was significantly correlated with chest pain and palpitations recovery (AUC, i.e., area under the curve = 0.66, CI [0.57– 0.75], *p* = 0.001 and AUC = 0.60, CI [0.51– 0.69], *p* = 0.03, respectively).

**Conclusion:**

Sulodexide significantly improves long-lasting post-COVID-19 endothelial dysfunction and alleviates chest pain and palpitations.

## Introduction

The severe acute respiratory syndrome coronavirus 2 (SARS-CoV-2) was detected in China in December 2019 (1, 2). To date, more than 269 million people have been infected worldwide, and over 5 million people have died from the coronavirus disease 2019 (COVID-19) (3). The COVID-19 is a multisystem disease due to in part to the vascular endothelium injury (4, 5).

Beyond pulmonary injury, inflammation, and particularly cytokine storm, prothrombotic state and endothelial dysfunction are proved to be the main causes of detrimental outcome of patients with COVID-19 in the acute phase (6).

The new symptoms persisting 30 days after the onset of COVID-19, not explained by an alternative diagnosis, are frequently reported and are recognized by NICE guidelines as a “long-COVID-19 syndrome” (7). These symptoms are sometimes disabling and affect patient's quality of life and delay return to work (8). Besides the socioeconomic impact, the medical community is still not fixed on the possible causes, outcomes, and how to manage such patients. The non-understanding of patients by their doctors causes an increasingly perceived loss of confidence. Many recent reports raised the concern of long-lasting inflammation and endothelial dysfunction several months beyond the acute phase (9). In the cohort of 798 patients with long-COVID-19 from the TUN-EndCOV study, we found that about half (49.7%) of patients still have microcirculation impairment with a significant independent link between non-respiratory long-COVID-19 symptoms (chest pain, fatigue, and neurocognitive symptoms) and endothelial dysfunction in multivariate analysis (10). **“ESC group for atherosclerosis and vascular biology”** stated that it seems relevant to follow the endothelial function in convalescent patients for early detection and prevention of long-term cardiovascular complications (4). Endothelial cells dysfunction should be overlooked as a therapeutic target during acute and long COVID-19 (11, 12).

Long-COVID-19 management guidelines has been developed jointly by NICE, the Scottish Intercollegiate Guidelines Network (SIGN), and the Royal College of General Practitioners (RCGP) (7). These guidelines focused on ruling out organic diseases otherwise rehabilitation and psychological support are proposed. “What pathophysiological mechanisms underlie the most common presentations of post-COVID-19 syndrome?” is stated as a research recommendation (6, 9).

Sulodexide, a highly purified mixture of glycosaminoglycan that includes fast-moving heparin and dermatan sulfate, had beneficial effects on the fibrinolytic system, platelets, endothelial cells, and inflammation (13). Sulodexide used in the early stages of COVID-19 was associated with a limiting disease progression, a decreased need for oxygen support, and hospital care (14).

Sulodexide was not tested in the setting of long-COVID-19 syndrome. We hypothesized that it may be effective in the long-COVID-19 spectrum and particularly in the subset of symptoms linked to microcirculation impairment and endothelium dysfunction.

## Materials and Methods

### Study Design

This is a prospective multicenter quasi-experimental case–control study. The recruiting period extended from 20 January to 26 June 2021. The study protocol was recorded in the Pan African Clinical Trials Registries (PACTR) with trial ID PACTR202102867544936. The study had the local Ethics and Investigation Committee approval, being designated with approval number CPP SUD 0299/2020. All authors reviewed the manuscript for the accuracy and completeness of the data.

### Patients

**Figure 1:**
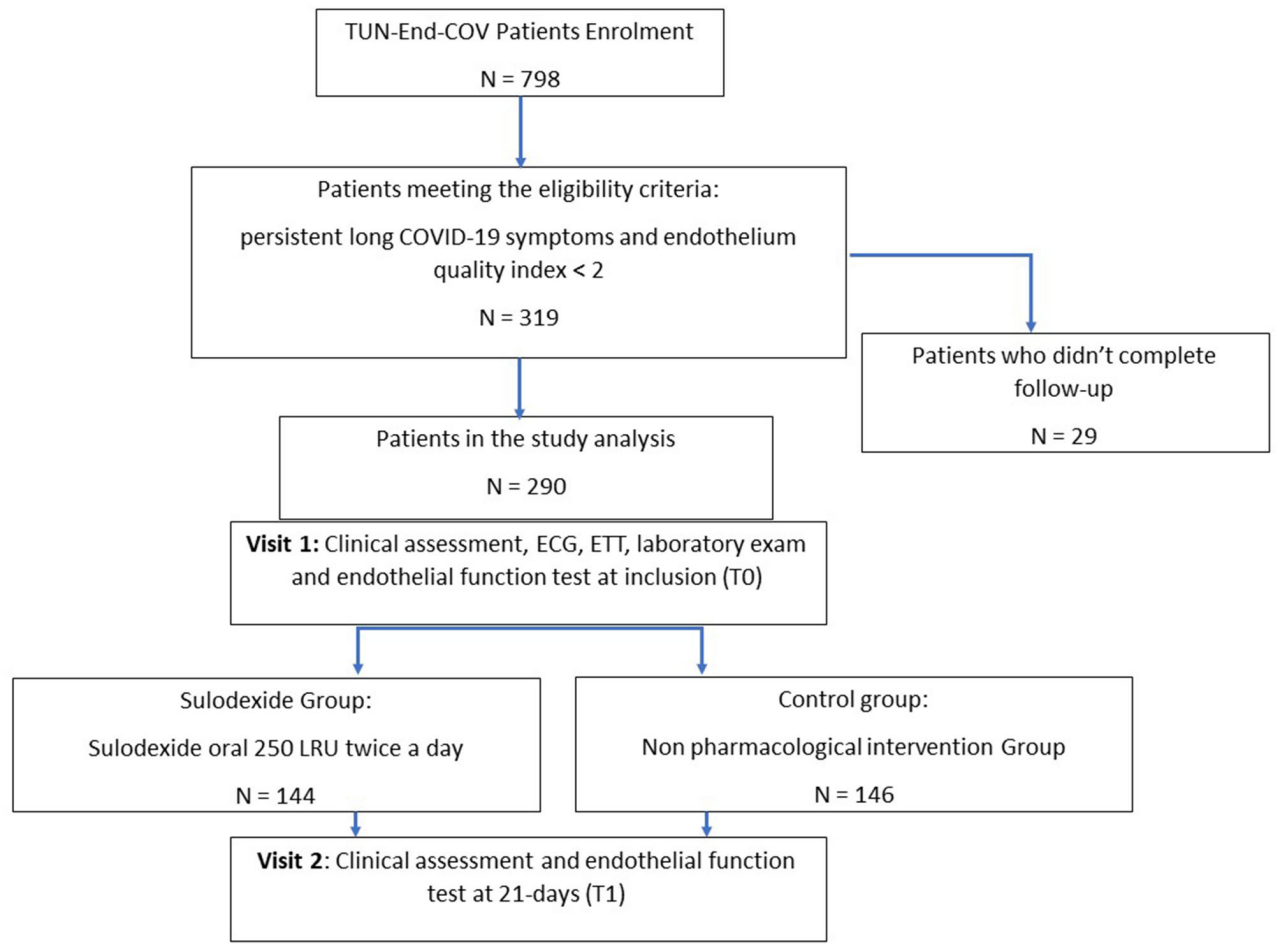
The study flow chart.

Patients were recruited by local health authorities relying on COVID-19 registry. The standard protocol of endothelial function study was previously detailed (10). After informed consent, all the eligible patients above 18 years old, with a recent diagnosis of COVID-19 infection in the past 1 to 6 months, confirmed by reverse-transcriptase polymerase chain reaction (RT-PCR) of nasopharyngeal swabs or viral rapid test, were enrolled. The symptomatic patients with proven endothelial dysfunction [endothelial quality index (EQI) < 2], assessed by a post-occlusive reactive hyperemia (PORH) finger thermal monitoring (E4-diagnose, Polymath Company, Tunisia) (15), were assigned into two groups. The sulodexide group received sulodexide (Vessel, AlfaSigma, Italy) 250 LSU (lipasemic units, approximately equivalent to 25 mg) two times a day during 21 days. The control group adhered to the study protocol without any medical treatment (Figure 1).

Main exclusion criteria were the patients recovered from COVID-19 with a good endothelial function (EQI ≥ 2), patients under anticoagulation or having experienced venous thromboembolism in the past 6 months, patients with chronic use of steroid medication, patients with life expectancy <1 year, according to clinical judgment, patient with symptoms related to an organic cause [acute coronary syndrome (ACS), pericarditis, heart failure, pulmonary embolism, pulmonary disease, etc.], pregnancy and breastfeeding and foreseen inability to attend scheduled visits. Patients who did not attend the scheduled 21-day visit were excluded from the data analysis.

The long-COVID-19 symptoms were screened relying on long-COVID-19 NICE guidelines (7). Electrocardiogram (ECG), transthoracic echocardiogram (TTE), laboratory examinations, and endothelial function test were done in the same initial consultation (T0). Objective symptoms evaluation was performed according to clinical scores. Shortness of breath was defined by a New York Heart Association class of dyspnea ≥ 2 (17). Fatigue was evaluated according to the modified fatigue severity scale (18). We used the Mini-Mental State Examination (MMSE) to assess cognitive performances. Cognitive difficulties were defined by an MMSE score < 24 (19).

Ambulatory 24-h cardiac monitoring DMS 300-4L was performed only in patients with palpitations. At ECG analysis, elevated heart rate (HR) was defined by an HR > 100 beats at rest, and supraventricular premature beats were defined by ectopic beats with narrow QRS (QRS ≤ 120 ms) and abnormal P wave and ventricular premature beats by ectopic beats with large QRS (QRS > 120 ms) and not preceded by P waves (20). Holter analysis Software was Cardioscan Premier 12. At Holter monitoring, the isolated ventricular ectopics which were less than 10% and the isolated supraventricular episodes which were less than 10% were regarded as insignificant arrhythmias.

A second visit with another clinical assessment and a second endothelial function test were performed in all patients at 21 days after inclusion (T1).

### Study End Points

Patient outcomes were assessed at 21 days after inclusion. Primary end points were: long-COVID-19 symptoms evolution from T0 to T1 and endothelial function outcome (delta EQI) defined as the difference between EQI at 21-day follow-up and EQI at inclusion. Delta EQI = EQI(T1)–EQI (T0).

### Statistical Analysis

Statistical analyses were performed using SPSS, Chicago (Statistical Package for the Social Sciences) version 23.0. The complete database is maintained by the study team.

Since we included a large long-COVID-19 population, we calculated a mean EQI amelioration without medical intervention of 0.2 (0.6) in an initial sample of patients. We then anticipated a 60% power of EQI after medical intervention, and we calculated a sample size of 142 subjects in each group.

Categorical variables were expressed as frequencies and percentages. For quantitative variables, we checked the normality of the distribution by the Kolmogorov–Smirnov test and the Shapiro–Wilk test. An estimate of the means with their standard deviations (SDs) and of the median with interquartile range (IQR) was thus carried out.

The comparison between two categorical variables was carried out by the Pearson's chi-squared test test or the Fisher's exact test if the conditions were not verified.

The Student's *t-*test was used for the comparison of two means when the distribution was Gaussian and by the non-parametric *U* test of Mann–Whitney or Kruskal–Wallis when the distribution was not Gaussian.

The McNemar's test was used to determine if there were differences in paired data during the follow-up in the different study groups.

The area under the curve (AUC) using the receiver operating characteristic curves determined the correlation between the delta EQI and long-COVID-19 symptoms recovery.

Estimates of risk ratios were presented with 95% confidence intervals (CIs). The value of *p* < 0.05 was considered statistically significant.

## Results

**Figure 2:**
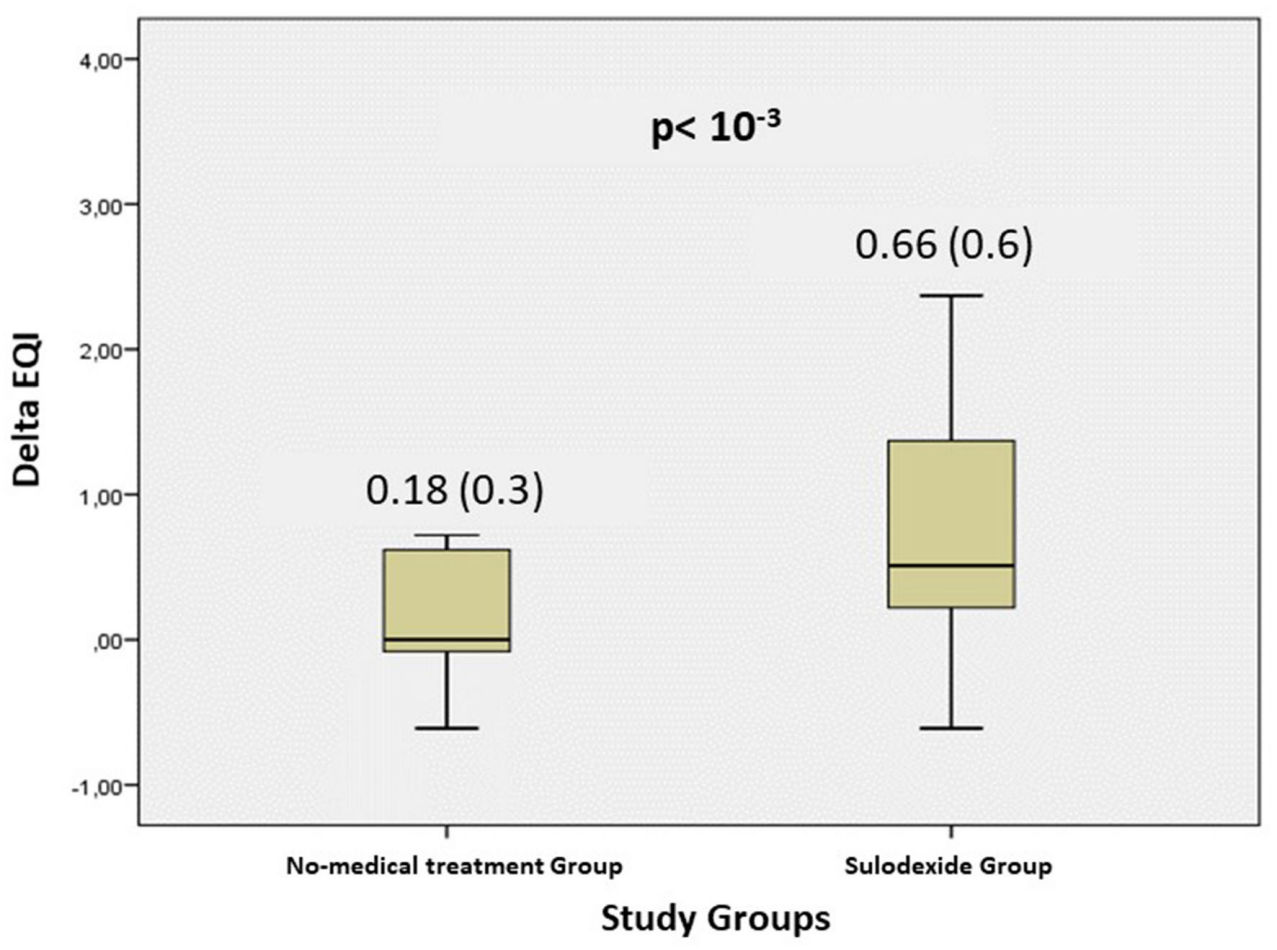
Evolution of the endothelium quality index (delta EQI) at 21-days follow-up in the 2 study groups.

**Figure 3:**
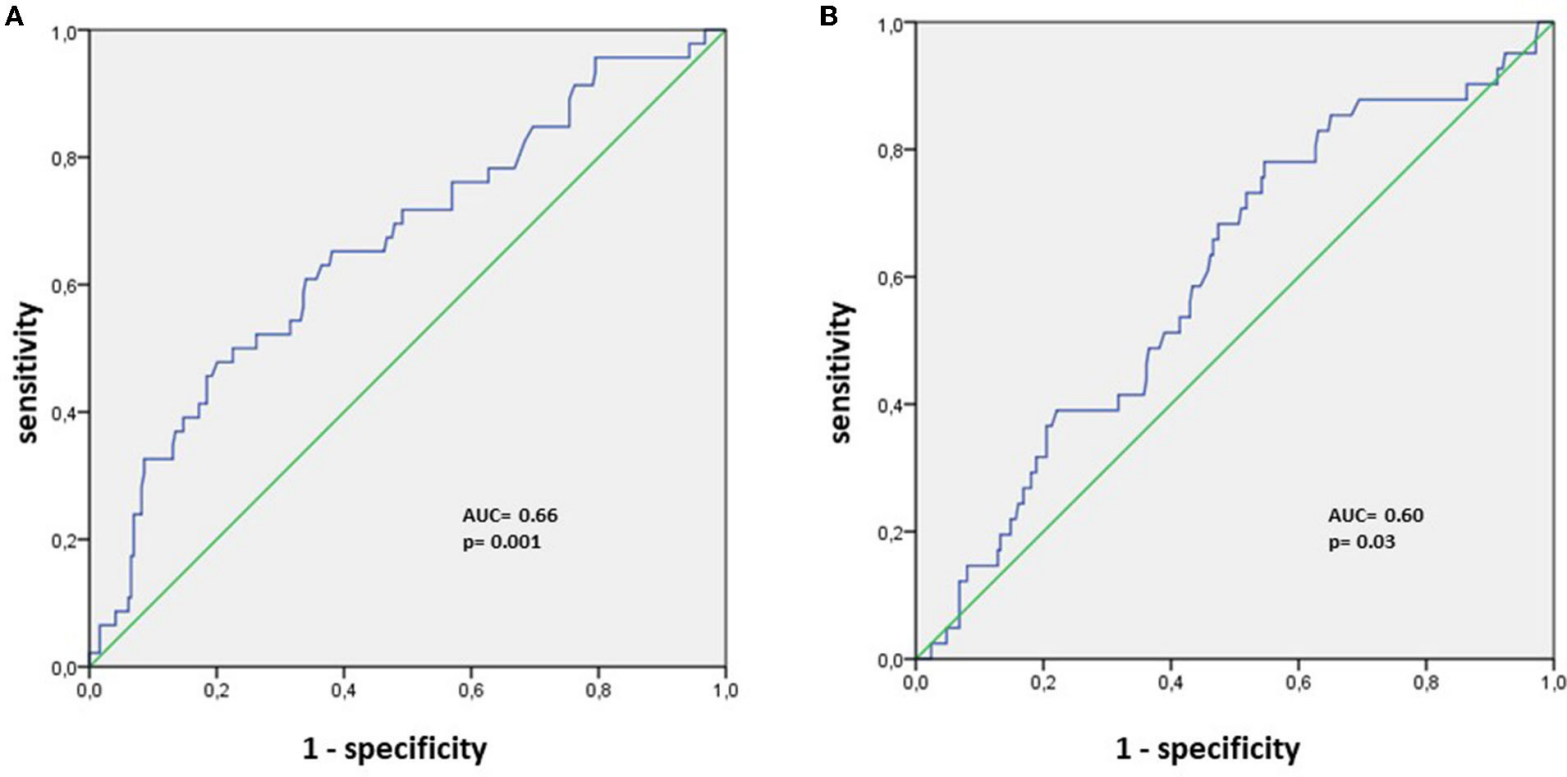
ROC analysis for endothelial function amelioration and **(A)** chest pain recovery, **(B)** palpitations recovery.

A total of 290 patients were included in this study at an average time of 70.81 (41.9) [28–180] days. They were assigned to a sulodexide group (144 patients) or a control group (146 patients). The demographics, clinical characteristics, and the number of chronic medications used by the patients were similar in the two study groups (Table 1). The median age was 54.15 (12.8) years. Females accounted for 54.1% of the participants (157 of 290). Hypertension was the most common chronic health condition, reported in 36.9% (107 of 290) and followed by diabetes 27.9% (81 of 290). The severity of COVID-19 infection, the extent of lesions at initial thoracic CT scan, and the average time between COVID-19 infection and inclusion were similar in the two groups (Table 1). The ECG analysis revealed an elevated HR at rest and premature supra ventricular and ventricular beats in 37 (12.8%), 13 (4.4%), and 14 (4.8%) of patients, respectively. Only 61 (21%) patients had ambulatory 24-h cardiac monitor. Holter monitoring analysis showed that among these patients, 19 (31.1%), 9 (14.8%), and 4 (6.6%) had sinus tachycardia, significantly isolated supra ventricular episodes, and ventricular ectopic beats, respectively. The rates of elevated HR at rest and premature beats were similar in the two groups (Table 1). The median troponin was 1.8 (2.3) ng/L. All patients had normal range troponin. The left ventricle ejection fraction (LVEF) in echocardiography was normal and similar in the two groups and there was no pericardial effusion (Table 1). Among the long-COVID-19 symptoms, fatigue was the most common symptom, reported in 54.5% (158 of 290), followed by shortness of breath reported in 53.8% (156 of 290) and chest pain 28.3% (82 of 290) (Table 1). The long-COVID-19 symptoms were similar in the two study groups (Table 1). Long-COVID-19 symptoms improved significantly at the 21-day follow-up in the 2 study groups (Table 2). At 21 days, the sulodexide group presented significantly less chest pain (83.7 vs. 43.6%, *p* < 10^−3^) and palpitations (85.2 vs. 52.9%, *p* = 0.009) than controls (Table 2). There was a trend of a significant decrease in fatigue (82.5 vs. 70.5%, *p* = 0.07) and neurocognitive disorders (78.9 vs. 53.8%, *p* = 0.08). The sulodexide group presented also a significant endothelial function amelioration compared to control group [median delta-EQI 0.66 (0.6) vs. 0.18 (0.3); *p* < 10^−3^] (Figure 2). Endothelial function improvement was significantly correlated with chest pain and palpitations recovery (AUC = 0.66, CI [0.57–0.75], *p* = 0.001 and AUC = 0.60, CI [0.51–0.69], *p* = 0.03, respectively) (Table 3, Figure 3).

**Table 1:** Baseline characteristics of the study population.

	**Total population (*N =* 290)**	**Sulodexide Group (*N =* 144)**	**No medical treatment Group (*N =* 146)**	***p*-value**
**Demographics and Clinical characteristics**	
Age (years) (mean, SD)	54.15 (12.8)	55.36 (12.9)	52.97 (12.6)	0.11
BMI (kg/m2) (median, IQR)	28.6 (4.5)	28.7 (2.4)	28.4 (2.8)	0.77^*^
Females (*n*, %)	157 (54.1)	73 (50.7)	84 (57.5)	0.24
Diabetes (*n*, %)	81 (27.9)	44 (30.6)	37 (25.3)	0.32
Hypertension (*n*, %)	107 (36.9)	58 (40.3)	49 (33.6)	0.23
Dyslipidemia (*n*, %)	45 (15.5)	25 (17.4)	20 (13.7)	0.38
Smoking (*n*, %)	20 (6.9)	7 (4.9)	13 (8.9)	0.17
Heart failure (*n*, %)	5 (1.7)	3 (2.1)	2 (1.4)	0.64
Coronary heart disease (*n*, %)	17 (5.9)	12 (8.3)	5 (3.4)	0.07
Pulmonary disease (*n*, %)	14 (4.8)	6 (4.2)	8 (5.5)	0.60
**Chronic medications before trial**
Aspirin (*n*, %)	21 (7.2)	13 (9.0)	8 (5.5)	0.24
RAAS Blockers (*n*, %)	86 (29.7)	46 (31.9)	40 (27.4)	0.39
Bblockers (*n*, %)	45 (15.5)	28 (19.4)	17 (11.6)	0.06
Statins (*n*, %)	60 (20.7)	35 (24.3)	25 (17.1)	0.13
**Severity of COVID 19 infection**
Moderate or severe symptoms (need of oxygen) (*n*, %)	107 (36.9)	56 (38.9)	51 (34.9)	0.48
**Extend of lesions at thoracic CT scan**
≥50 % (*n*, %)	29 (10)	13 (44.8)	16 (55.2)	0.58
**Delay between COVID-19 infection and inclusion (days)**	70.81 (41.9)	72.87 (41.1)	68.77 (42.7)	0.40
**Electrocardiogram**
Heart rate (mean, SD)	77.45 (11.3)	78.93 (12.4)	76.31 (10.3)	0.15
Heart rate at rest > 100 (*n*, %)	37 (12.8)	18 (12.5)	19 (13.0)	0.89
Premature supra ventricular complexes (*n*, %)	13 (4.4)	6 (4.1)	7 (4.8)	0.84
Premature Ventricular complexes (*n*, %)	14 (4.8)	7 (4.9)	7 (4.8)	0.97
**Troponin** (ng/L) (median, IQR)	1.8 (2.3)	1.6 (3.3)	1.85 (1.7)	0.69^*^
**Ambulatory cardiac monitor** (n, %)	61 (16)	27 (18.8)	34 (23.3)	0.34
**Echocardiographic parameters**
LVEF (%) (median, IQR)	61 (4)	61 (2.5)	61.5 (4)	0.64^*^
Pericardial effusion (*n*, %)	0	0	0	-
**Long covid symptoms**				
Fatigue (*n*, %)	158 (54.5)	80 (55.6)	78 (53.4)	0.71
Chest pain (*n*, %)	82 (28.3)	43 (29.9)	39 (26.7)	0.55
Palpitations (*n*, %)	61 (16)	27 (18.8)	34 (23.3)	0.34
Shortness of breath (*n*, %)	156 (53.8)	71 (49.3)	85 (58.2)	0.12
Cough (*n*, %)	45 (15.5)	23 (16.0)	22 (15.1)	0.83
Headaches (*n*, %)	76 (26.2)	31 (21.5)	45 (30.8)	0.07
Gastro-intestinal syndrome (*n*, %)	25 (8.6)	8 (5.6)	17 (11.6)	0.06
Anosmia (*n*, %)	11 (3.8)	3 (2.1)	8 (5.5)	0.13
Neuro-cognitive difficulties (*n*, %)	45 (15.5)	19 (13.2)	26 (17.8)	0.27

*BMI, body mass index; EQI, endothelium quality index; LVEF, left ventricular ejection fraction; LVGLS, left ventricular global longitudinal strain; RAAS, renin angiotensin aldosterone system*.

* *Mann–Whitney U test*.

**Table 2:** Evolution of the long-COVID-19 symptoms in the study groups.

	**Sulodexide group (*****N*** **=** **144)**				**No medical treatment group (*****N*** **=** **146)**				***p*-value ^†^ % recovery**
	**T0**	**T1**	*p*-value^*^	**% Recovery**	**T0**	**T1**	*p*-value^*^	**% Recovery**	
Chest pain (*n*, %)	43 (29.9)	7 (4.9)	**<0.001**	36/43 (83.7)	39 (26.7)	19 (13)	**<0.001**	20/39 (43.6)	**<10** ^**−3**^
Palpitations (*n*, %)	27 (18.8)	4 (2.7)	**<0.001**	23/27 (85.2)	34 (23.3)	16 (10.9)	**<0.001**	18/34 (52.9)	**0.009**
Fatigue (*n*, %)	80 (55.6)	14 (9.7)	**<0.001**	66/80 (82.5)	78 (53.4)	23 (15.8)	**<0.001**	55/78 (70.5)	0.07
Shortness of breath (*n*, %)	71 (49.3)	20 (13.9)	**<0.001**	51/71 (71.8)	85 (58.2)	32 (21.9)	**<0.001**	53/85 (62.4)	0.21
Cough (*n*, %)	23 (16.0)	4 (2.8)	**<0.001**	19/23 (82.6)	22 (15.1)	8 (5.5)	**<0.001**	14/22 (63.6)	0.15
Headaches (n, %)	31 (21.5)	7 (4.9)	**<0.001**	24/30 (80)	45 (30.8)	16 (11)	**<0.001**	29/45 (64.4)	0.14
Gastro-intestinal syndrome (*n*, %)	8 (5.6)	1 (0.7)	**0.01**	7/8 (87.5)	17 (11.6)	5 (3.4)	**<0.001**	12/17 (70.6)	0.35
Anosmia (*n*, %)	3 (2.1)	1 (0.7)	0.5	2/3 (66.7)	8 (5.5)	2 (1.4)	**0.03**	6/8 (75)	0.78
Neuro-cognitive difficulties (*n*, %)	19 (13.2)	4 (2.8)	**<0.001**	15/19 (78.9)	26 (17.8)	12 (8.2)	**<0.001**	14/26 (53.8)	0.08

*ACS, acute coronary syndrome; T0, Symptoms' assessment at inclusion; T1, symptoms' assessment at 21-day follow-up*.

* *McNemar's test*;

† *chi-squared test*.

*Bold values refer to significant p-value*.

**Table 3:** Correlation between endothelial function improvement and long-COVID-19 symptoms recovery.

	**AUC**	**CI**	***p*-value**
Chest pain	0.66	[0.57–0.75]	**0.001**
Palpitations	0.60	[0.51–0.69]	**0.03**
Fatigue	0.52	[0.45–0.59]	0.47
Shortness of breath (dyspnea)	0.49	[0.42–0.56]	0.83
Cough	0.54	[0.44–0.55]	0.35
Headaches	0.57	[0.50–0.65]	0.07
Gastro-intestinal syndrome	0.51	[0.38–0.64]	0.82
Anosmia	0.39	[0.20–0.58]	0.30
Neuro-cognitive difficulties	0.53	[0.43–0.63]	0.55

*AUC, Area under the curve; CI, confidence interval*.

## Discussion

Our findings provide evidence that sulodexide significantly improves long-lasting post-COVID-19 endothelial dysfunction and alleviates chest pain and palpitations.

In our study, we found that fatigue, shortness of breath, and chest pain were the most reported post-COVID-19 sequalae. Long-COVID-19 syndrome was characterized by a combination of non-specific symptoms. Cardiovascular (CV) symptoms especially chest pain and palpitations were frequently reported (16, 21–23).

The underlying pathophysiology of CV symptoms is not yet well defined. Several explanations have been proposed, for example, a microvascular and endothelial dysfunction (24–26), vascular pericyte involvement (27), a systemic and myocardial inflammation (28, 29), an immune dysregulation, and a nervous system autonomic instability (30).

To date, only limited series of *in-vitro* trials were available and there were no published *in-vivo* trials in the long-COVID 19 setting.

In a previous publication of TUN-EndCOV, we found that about half (49.7%) of patients still have microcirculation impairment monitored by a non-invasive PORH finger thermal device. Endothelial dysfunction (EQI < 2) was significantly related to non-respiratory long-COVID 19 symptoms (chest pain, fatigue and neurocognitive symptoms) in multivariate analysis (OR = 1.522 (1.072–2.160), *p* = 0.019) (10). These findings suggest that persistent microvascular and endothelial dysfunction could explain a subset of long-COVID-19 symptoms. Another study investigated *in-vivo* vascular endothelial function with a non-invasive peripheral arterial tonometry (Endopat, Itamar). In 3 matched series, flow-mediated dilation of peripheral arteries was substantially lower in patients with post-COVID-19 when compared to acute infection and controls (9).

These findings support the evidence of increased rates of vascular complications in patients with post-COVID-19 compared to general population and the necessity of effective treatment to avoid such complications (31). The increasing number of symptomatic patients with long COVID-19 looking for an effective solution is nowadays a real health public problem. NICE guidelines focused on ruling out organic diseases otherwise rehabilitation and psychological support are proposed (7). Betablockers and ivabradine were proposed in patients with post-orthostatic tachycardia syndrome (POTS) (32). Otherwise, statins, oral anticoagulants, and antidepressors were suggested and widely used in daily practice without sufficient evidence (32).

In a review about the pathogenesis of COVID-19 through the lens of an undersulfated and degraded epithelial and endothelial glycocalyx, the prevention and treatment protocols proposed were to preserve and repair epithelial and endothelial glycocalyx integrity (33). This objective seems to be relevant to the patients with post-COVID-19 with persistent glycocalyx damage and endothelial dysfunction (34, 35).

Since endothelial dysfunction seems to be a serious driving cause of non-respiratory long-COVID-19 symptoms, we hypothesized that targeting microvascular impairment could be an effective solution. In this quasi-experimental TUN-EndCOV study, we have selected sulodexide (Vessel, AlfaSigma, Italy) 250 LSU two times a day during 21 days for symptomatic patients with proven endothelial dysfunction (EQI < 2). A clinical assessment and endothelial function test were performed in all patients at inclusion and at 21 days later.

Sulodexide, an orally administered highly purified mixture of glycosaminoglycan that includes fast-moving heparin and dermatan sulfate, had beneficial effects on the fibrinolytic system, platelets, endothelial cells, and inflammation (13). Sulodexide is known by its pleotropic action and protective effect on the endothelium (36, 37). It presents antithrombotic properties through reduction of fibrinogen (38, 39) and plasminogen activator inhibitor-1 (PAI-1) (38, 40) and is thought to have anti-inflammatory properties (39–42). Across different cardiovascular indications, the use of sulodexide was associated with reduced risk of venous thromboembolism, myocardial infarction, cardiovascular mortality, and all-cause mortality (43, 44). Despite the potential interest, limited data exist about the safety and efficacy of sulodexide in patients with COVID-19 (45, 46). A randomized study supports the effectiveness of sulodexide in mitigating the severe clinical progression rate of COVID-19, compared to the prevalent standard of care, when used in the early symptomatic stages of the disease by decreasing the need for hospital admission, oxygen requirements, and the serum levels of inflammatory and prothrombotic markers (14). These findings, especially lower inflammatory and prothrombotic markers, could explain faster endothelium restoration after a COVID-19 infection.

Our results support evidence that sulodexide significantly improves long-lasting post-COVID-19 endothelial dysfunction and alleviates related chest pain (83.7%, *p* < 10^−3^) and palpitations (85.2%, *p* = 0.009). There was also a trend of a significant decrease in fatigue (82.5%, *p* = 0.07) and neurocognitive disorders (78.9%, *p* = 0.08).

TUN-EndCOV trial is not only the largest *in-vivo* trial that has proved that non-respiratory long-COVID-19 symptoms are related to endothelial dysfunction but is also the first to prove that targeting these symptoms by sulodexide could be an effective solution for more than 80% of patients within a short-time medication of 3 weeks. Non-respiratory symptoms recovery was in parallel with endothelial function restoration. TUN-EndCOV is a real-life study, in which patients with cofactors of endothelial dysfunction (diabetes, hypertension, age, obesity, etc.) were not under represented as it could be in a randomized study. Our findings support the emphasized patients' hope in regaining their wellbeing and accelerating their return to work. The synergic activity of sulodexide's components and its pleiotropic effects may be essential to reach such results.

### Limitations and Perspectives

The main limitations of the study:

- This was a quasi-experimental study with a control group. The need of placebo-randomized study is warranted to confirm such results.
- Further studies with larger population are needed to confirm the significant recovery of the other “non-respiratory” symptoms such as neurocognitive difficulties and fatigue.
- Limited follow-up of 3 weeks. Long-term follow-up is needed for major events (mortality, thromboembolic complications, stroke, myocardial injury, etc.) analysis.
- Only sulodexide with one-dose regimen has been tested. Further studies with other protocols and drugs targeting endothelial dysfunction must be conducted.
- Only symptomatic patients with proven endothelial dysfunction were included which raises the concern of need and availability of such tests in clinical practice.

## Conclusion

Long-lasting microcirculation and endothelial dysfunction have been proved *in-vitro* and *in-vivo* studies in convalescent patients with COVID-19. Non-respiratory symptoms may be related to microcirculation impairment. Our results support that targeting this cause with sulodexide accelerates patients' recovery in parallel with vascular endothelium restoration. Other clinical trials are warranted.

## Data Availability Statement

The raw data supporting the conclusions of this article will be made available by the authors, without undue reservation.

## Ethics Statement

The studies involving human participants were reviewed and approved by the study had the local Ethics and Investigation Committee approval, being designated with approval number CPP SUD 0299/2020. Written informed consent for participation was not required for this study in accordance with the national legislation and the institutional requirements.

## Author Contributions

LA, SA, SC, HI, and JJ conceived and designed the study, had full access to all of the data in the study, and take responsibility for the integrity of the data and the accuracy of the data analysis. SC, SA, JJ, and LA drafted the paper and performed the analysis. ST, SK, AG, ZM, and YT contributed to data collection and interpretation. All authors critically revised the manuscript for relevant intellectual content and gave final approval for the version to be published. All authors agree to be accountable for all aspects of the work and will answer any questions related to the accuracy or integrity of the work.

## Conflict of Interest

The authors declare that the research was conducted in the absence of any commercial or financial relationships that could be construed as a potential conflict of interest.

## Publisher's Note

All claims expressed in this article are solely those of the authors and do not necessarily represent those of their affiliated organizations, or those of the publisher, the editors and the reviewers. Any product that may be evaluated in this article, or claim that may be made by its manufacturer, is not guaranteed or endorsed by the publisher.
